# Obstetrics and Gynecology Medicine: Go from Bench to Bedside

**DOI:** 10.3390/life15020233

**Published:** 2025-02-05

**Authors:** Stefano Canosa, Anna Maria Nuzzo

**Affiliations:** 1 IVIRMA Global Research Alliance, LIVET, 10126 Turin, Italy; 2 Department of Surgical Sciences, Gynecology and Obstetrics 2, City of Health and Science-S. Anna University Hospital, University of Turin, 10126 Turin, Italy; a.nuzzo@unito.it

## 1. Recalling the Special Issue Aims and Scope

As we reach the conclusion of this Special Issue, Obstetrics and Gynecology Medicine: Go From Bench to Bedside, it is evident that the dynamic intersection between basic science and clinical practice continues to drive the evolution of care in women’s health. Over the last few decades, we have witnessed remarkable advancements in the understanding of reproductive biology, pregnancy complications, and gynecologic disorders. From the molecular mechanisms underlying endometriosis to breakthroughs in fertility preservation, these developments have reshaped the landscape of obstetrics and gynecology. Yet, despite significant strides, there remain critical gaps in our knowledge. We continue to face challenges in understanding the full etiology of many common conditions and how to treat them. Consequently, translating discoveries from the laboratory into widespread clinical applications remains a persistent obstacle, hindering the development of personalized treatments for patients. This Special Issue has sought to address some of these gaps by highlighting cutting-edge research that bridges the divide between basic science and clinical application. Through a curated collection of studies, we have explored innovative approaches in areas such as diagnoses, obstetrics, gynecological cancers, placental pathology, pregnancy loss, and assisted reproduction. These contributions underscore the importance of collaborative, interdisciplinary research in shaping the future of obstetrics and gynecology.

## 2. An Overview of Published Articles

The contributions to this Special Issue are listed in the section *List of Contributions* and are hereafter briefly overviewed.

The first contribution, from Elekes T et al. (Contribution 1), assessed the learning curve and the effectiveness of first-trimester fetal cardiovascular ultrasound screenings, providing that the curve may reach as high as 90% in terms of major congenital heart defects from an unselected pregnant population.

The second contribution, from Guerra de Melo I et al. (Contribution 2), investigated the impact of endothelial dysfunction (ED)-related genes and single-nucleotide polymorphisms (SNPs) on ovarian cancer (OC)-related venous thromboembolism (VTE) and patient thrombogenesis-independent prognosis. The authors observed that *NOS3* upregulation was linked to lower VTE incidence, while *SELP* upregulation was associated with shorter overall survival. Dismissing patients with VTE before OC diagnosis, *SELP rs6136 T* allele carriers presented lower progression-free survival.

The third contribution, from Rolfo A et al. (Contribution 3), investigated the impact of COVID-19 vaccination on third-trimester placental antioxidant defense markers. They observed that SARS-CoV-2 infection induced placental oxidative stress (OxS), which is countered by a placental adaptive antioxidant response. Vaccination during pregnancy enhanced the placental defense, further supporting the safety and benefits of the COVID-19 vaccination in preventing complications and protecting fetal development.

The fourth contribution, from Cimadomo D et al. (Contribution 4), applied artificial intelligence to time-lapse microscopy to picture the association between embryo and inner-cell-mass (ICM) area, the ICM/Trophectoderm ratio, and the zona pellucida thickness at sequential blastocyst expansion stages, with (i) euploidy and (ii) live-birth per transfer. A quantitative standardized expansion assay (qSEA) was developed, outlining that faster and more consistent zp thinning processes can be observed among euploid blastocysts implanted versus not implanted blastocysts. qSEA can represent a promising objective, quantitative, and user-friendly strategy to predict embryo competence.

A fifth contribution, from Coca R et al. (Contribution 5), evaluated the status of *HER2* overexpression among new cases of breast neoplasia with an impact on the natural history of breast cancer disease and therapeutic personalization according to staging. Anti-*HER2* therapy in any therapeutic stage has shown increased efficiency in blocking tyrosine kinase receptors, evidenced by the high percentage of complete pathological responses, as well as the considerable percentage of complete remissions and stationary disease, in relation to the HER2-positive patient group.

A sixth contribution, from Cheng C et al. (Contribution 6), aimed to determine whether diabetes mellitus (DM) increases the risk of pregnancy loss and to identify other potential risk factors. The study concluded that DM together with older age, gynecological disorders, and inflammation disorders represent factors for greater risk of experiencing pregnancy loss. Healthcare providers should proactively manage and educate diabetic patients to reduce their risk of pregnancy loss.

A seventh contribution, from Delgado-Miguel C et al. (Contribution 7), aimed to determine the diagnostic value of clinical, ultrasound, and inflammatory laboratory markers in pediatric ovarian torsion (OT). It was established that the neutrophil-to-lymphocyte ratio (NLR) can be considered as a useful predictor of pediatric OT in cases with clinical and ultrasound suspicion. Values above 2.57 may help to anticipate urgent surgical treatment in these patients.

An eighth contribution, from Vrachnis D et al. (Contribution 8), investigated the associations of the amniotic fluid angiotensinogen of the renin–angiotensin system with fetal growth abnormalities. Multiple regression analysis revealed a statistically significant negative correlation between the angiotensinogen levels and gestational age and a statistically significant positive correlation between the birth weight and angiotensinogen levels. These findings suggest that fetal growth abnormalities did not correlate with differences in the amniotic fluid levels of angiotensinogen in early second-trimester pregnancies. However, increased angiotensinogen levels were found to be consistent with a smaller gestational age at birth and increased BMI of neonates.

A ninth contribution, from Xue Y et al. (Contribution 9), aimed to establish an early-onset pre-eclampsia prediction model by clinical characteristics, risk factors, and routine laboratory indicators from pregnant women at 6 to 10 gestational weeks. They observed that the performance of 12 clinical risk factors alone (among them, rates of diabetes, antiphospholipid syndrome (APS), kidney disease, obstructive sleep apnea (OSAHS), primipara, history of pre-eclampsia, and assisted reproductive technology (ART)) in predicting early-onset PE is poor, and the performance significantly improved when combing risk factors with the other 38 routine laboratory indicators. The support vector machine (SVM) model showed the best AUC^ROC^, specificity, and sensitivity compared with a logistic regression model and decision tree model.

A tenth contribution, from Subramaniam V et al. (Contribution 10), aimed to determine the adverse maternal and fetal outcomes due to COVID-19 infection. Comparing early vs. severe stages of COVID-19 infection, it was shown that the severe-stage disease increased the risk of preterm birth and preterm birth before 34 weeks. The severe-stage disease also increased the Neonatal Intensive Care Unit (NICU) admission with lower birth weight. Moreover, the unvaccinated mothers had an increased risk of preterm birth before 34 weeks.

An eleventh contribution, from Dicu-Andreescu I et al. (Contribution 11), reviewed cases of abdominal parietal metastasis in recent decades, including a new case of a 4.5 cm abdominal parietal metastasis at the site of the scar of the former drain tube 28 months after the diagnosis of stage IIB cervical cancer (adenosquamous carcinoma), treated by external radiotherapy with concurrent chemotherapy and intracavitary brachytherapy and subsequent surgery (type B radical hysterectomy). The discussion explored the potential pathways for parietal metastasis and the impact of incomplete surgical procedures on the development of metastases.

A twelfth contribution, from Jung L et al. (Contribution 12), performed a literature search on cervical carcinoma and its relation to HPV infection. The diagnosis, the presence of HPV, and its role in cancer screening, together with the detection of new therapeutic options, were assessed across the history to continuously optimize cervical carcinoma diagnosis and treatment.

A thirteenth contribution, from Enache I et al. (Contribution 13), aimed to review the most relevant studies based on deep learning in ultrasound anomaly scan evaluation of the most complex fetal systems (heart and brain), which enclose the most frequent anomalies. AI can provide a more accurate and efficient method for identifying and diagnosing fetal anomalies, offering a promising avenue for future improvements in fetal healthcare.

A fourteenth contribution, from Flore A et al. (Contribution 14), describes the different types of hydatidiform moles and their subsequent mechanisms, which is useful to calculate the recurrence risk and estimate the method of progression to a malign form. This review synthesizes the heterogeneous mechanisms and their implications in genetic counseling.

A fifteenth contribution from Bartolacci A et al. (Contribution 15), provides a comprehensive review and proposed literature score of the effects of chemical and physical parameters on embryo developmental competence. Particularly, a prevalence of studies suggested a favor of the embryo culture performed at 37 °C and 5% oxygen. 

A sixteenth contribution from Kovács BP et al. (Contribution 16), developed a 70 min full body “reproductive gymnastics”, including strengthening, stretching, and relaxation exercises combined with yoga-inspired moves and diaphragmatic breathing with meditation elements to activate the parasympathetic pathway and stress relief. In this way, fertility can be improved through the combination of natural supplements and our targeted, moderate physiotherapy program in women with an age-related decline of ovarian reserve.

A seventeenth contribution, from Vida B et al. (Contribution 17), represents a systematic review and meta-analysis protocol aiming to provide insights into the optimal sentinel lymph node (SLN) detection method with potential implications for clinical practice guidelines in vulvar cancer management. 

An eighteenth contribution, from Psomiadou V et al. (Contribution 18), is a systematic review suggesting that solitary mediastinal metastasis from ovarian cancer is a very rare condition. Physicians should pay close attention when routinely evaluating thoracic scans from patients with ovarian malignancy as well as individualizing the management in such patients, since surgical resection can also be performed.

## 3. Discussion

Recent advances in translational research in obstetrics and gynecology have significantly impacted clinical practice, improved patient care, and introduced innovative approaches in the treatment of various conditions. Translational research plays a crucial role in bridging the gap between laboratory discoveries and their practical application in clinical settings. The rapid development of new diagnostic tools, therapeutic strategies, and improved clinical outcomes in this field can be attributed to the synergy between academic research and clinical practice, often supported by collaborations between public and private institutions.

### 3.1. Recent Developments

Research has led to a better understanding and treatment of gynecological cancers, including ovarian, cervical, and endometrial cancers. New therapies that target specific molecular pathways are improving treatment outcomes and minimizing side effects. The integration of targeted therapies and immunotherapies has been a notable achievement, especially for advanced cancers. Personalized medicine, based on genetic profiling, is another example where research has been translated into more effective, individualized treatment plans [1]. Research into the molecular mechanisms behind conditions linked to placental pathology has provided insights into potential diagnostic biomarkers and novel therapeutic approaches. For instance, the identification of specific biomarkers that could predict the onset of pre-eclampsia earlier allows for timely interventions that improve maternal and fetal outcomes [2]. The discovery of new biomarkers for diagnosing and predicting outcomes in obstetrics and gynecology is transforming patient care. In ovarian cancer, for example, novel biomarkers are being explored to improve early detection and prognosis [3]. The automation of assisted reproductive technologies, such as in vitro fertilization (IVF), has made great strides, thanks to translational research. New automated systems for embryo culture, genetic screening, and embryo selection based on genetic and phenotypic characteristics are improving success rates and reducing human error. Furthermore, artificial intelligence (AI) and machine learning models are now being used to predict the success of IVF treatments more accurately, offering patient-tailored treatment plans based on a deeper understanding of their individual circumstances [4]. Consequently, personalized medicine is emerging as a powerful tool in obstetrics and gynecology, with translational research uncovering genetic, epigenetic, and environmental factors that influence disease progression and treatment responses.

### 3.2. Gap in Knowledge

The process of transforming research innovations into new health products, as well as diagnostic and therapeutic strategies, remains a major issue of contemporary biomedical medicine due to several interconnected challenges. While significant advancements in scientific research have led to a deeper understanding of disease mechanisms and potential treatments, translating these findings into tangible, clinically effective products often takes years, if not decades. Addressing these hurdles—ranging from regulatory and financial obstacles to collaboration and accessibility issues—requires coordinated efforts among researchers, policymakers, industry leaders, and healthcare professionals.

### 3.3. Special Issue Results

The contributions included in this Special Issue provided significant findings across oncology, prenatal care, and reproductive health.

The care of cancer patients is an extremely important issue in modern medicine. Optimizing diagnostic and treatment protocols will ensure a better prognosis and quality of life for these patients. Recent advances in ovarian and breast cancer research have brought new hope for improved treatments and early detection. In ovarian cancer, researchers are making progress in identifying new genetic and molecular markers that will allow for earlier diagnoses and more precise targeting of therapies [5]. In this context, Guerra de Melo I et al. found that endothelial dysfunction-related genes and SNPs, in particular *NOS3* and *SELP*, significantly influenced the incidence of venous thromboembolism and overall survival in ovarian cancer patients, with the upregulation of certain genes correlating with better or worse outcomes (Contribution 2), In addition, Psomiadou V et al. highlighted the rarity of solitary mediastinal metastases in ovarian cancer, emphasizing the need for individualized treatment due to the unique challenges of this disease (Contribution 18). In breast cancer, ongoing research is focused on refining targeted therapies, particularly for specific subtypes such as *HER2*-positive and triple-negative breast cancer. New drug combinations, as well as innovations in immunotherapy and hormone therapy, are showing potential to improve outcomes and reduce resistance to treatment [6]. In our Special Issue, *HER2* overexpression in breast cancer was shown to be a critical factor in determining therapeutic strategies, with anti-*HER2* therapies showing high efficacy in reducing tumor progression (Contribution 5), Research into cervical cancer is currently focusing on the role of HPV (human papillomavirus) in the pathogenesis of the disease. On the treatment front, research is exploring new therapies, including targeted treatments and immunotherapies, to more effectively combat advanced cervical cancer [7]. The review published by Dicu-Andreescu I et al. discussed abdominal parietal metastasis in cervical cancer, noting that incomplete surgery may contribute to metastatic spread (Contribution 11), while Jung L et al. highlighted the importance of HPV in cervical cancer, advancing diagnostic and therapeutic strategies to optimize outcomes (Contribution 12). Vulvar cancer was another topic, as sentinel lymph node detection methods were reviewed with the aim of refining clinical guidelines for more accurate diagnoses and treatments (Contribution 17). Finally, Delgado-Miguel C et al. demonstrated that the neutrophil-to-lymphocyte ratio (NLR) could serve as an effective predictor of pediatric ovarian torsion, aiding in early diagnosis and intervention (Contribution 7).

Advances in prenatal screening are an example of translational medicine efforts, as the use of first-trimester fetal cardiovascular ultrasound screenings has been shown to detect up to 90% of major congenital heart defects (Contribution 1). In addition, the use of AI has demonstrated its potential in the detection of fetal anomalies, particularly heart and brain anomalies (Contribution 13). Biochemical markers such as amniotic fluid angiotensinogen levels also showed a significant correlation with both birth weight and gestational age, suggesting its potential role in understanding fetal growth (Contribution 8).

Placental pathology is the study of diseases or abnormalities that affect the placenta during pregnancy. The placenta is a vital organ that provides nutrients and oxygen to the developing fetus, removes waste products and plays a key role in hormone production. If the placenta is diseased or damaged, it can lead to complications for both mother and baby [8]. The protective effects of the COVID-19 vaccination during pregnancy were evaluated by Rolfo A et al. (Contribution 3), who showed that it enhanced placental antioxidant defenses and supported fetal development by counteracting oxidative stress induced by SARS-CoV-2. In line with these findings, Subramaniam V et al. found that severe COVID-19 infection during pregnancy was associated with an increased risk of preterm birth and neonatal intensive care unit admission, while the vaccination reduced these risks (Contribution 10),. In contrast, Xue Y et al. developed a predictive model for early-onset pre-eclampsia that significantly improved predictive accuracy when clinical risk factors were combined with laboratory indicators (Contribution 9).

Human embryo culture is a critical component of successful in vitro fertilization (IVF) in the field of assisted reproductive technology (ART). The process of human pre-implantation development is complex and requires a highly controlled environment to ensure success. During embryo culture, both chemical and physical factors are essential to support proper embryo growth and maintain viability [9]. Bartolacci A et al. identified optimal culture conditions for embryo development, suggesting that embryo culture at 37 °C and 5% oxygen improves outcomes (Contribution 15). In another area of research, the application of artificial intelligence (AI) to reproductive medicine has revolutionized the way healthcare professionals approach fertility, pregnancy, and related conditions. AI has been particularly influential in areas such as in vitro fertilization (IVF), where it helps to select and prioritize embryos, predict pregnancy success, and optimize the decision-making process. Cimadomo D et al. developed the qSEA AI tool, which can predict embryo implantation success based on specific characteristics such as zona pellucida thinning, providing a more reliable method for selecting competent embryos (Contribution 4). On another topic related to infertility, Kovács BP et al. proposed a 70 min exercise program combining physiotherapy and relaxation techniques to improve fertility, especially in women with age-related decline in ovarian reserve (Contribution 16). Cheng C et al. identified diabetes, advanced age, and gynecological and inflammatory disorders as major risk factors for pregnancy loss and emphasized the importance of managing these factors to reduce the risk (Contribution 6). Finally, Flore A et al. synthesized the mechanisms behind hydatidiform moles, providing important insights into recurrence risk and genetic counseling (Contribution 14).

These findings collectively contribute to advancements in diagnostic tools, therapies, and personalized care across a range of medical fields.

### 3.4. Future Work

Looking ahead, the path to further progress will require a focused effort in several key research areas. One priority is the need for more robust longitudinal studies to better understand how environmental and genetic factors interact across the lifespan to influence conditions such as endometriosis, infertility, and pre-eclampsia. Further investment is also needed in precision medicine, where individualized treatments based on genetic profiles and other biomarkers could significantly improve outcomes for women. Research in maternal–fetal medicine, particularly in areas such as immune modulation during pregnancy and placental function, offers exciting potential to advance treatment strategies. In addition, as we move from bench to bedside, the integration of technology—such as artificial intelligence and machine learning—into obstetrics and gynecology will offer new opportunities for early detection, diagnosis and personalized care. Ensuring equitable access to these innovations, especially for underserved populations, will be critical to addressing disparities in maternal and reproductive health outcomes.

## 4. Conclusions

Translational research is transforming obstetrics and gynecology, making it an exciting and rapidly evolving field. From new cancer treatments to stem cell therapies and automated IVF, basic science is being translated into practical applications that improve patient outcomes. The collaboration between academia, clinical practice, and industry is essential to ensure that these innovations reach the bedside efficiently, ultimately improving patient care and quality of life for women worldwide. In conclusion, while we have made significant progress in the field of obstetrics and gynecology, the journey from bench to bedside is still a work in progress. The future of women’s health will be shaped by continued interdisciplinary collaboration, innovation, and a commitment to translating scientific discoveries into tangible, life-changing clinical applications. As researchers, clinicians, and policymakers, we must prioritize these areas to ensure that every woman benefits from the advances that lie ahead.
